# Two-Dimensional Nanoporous Cross-linked Polymer Networks as Emerging Candidates for Gas Adsorption

**DOI:** 10.1021/acsomega.3c09042

**Published:** 2024-03-22

**Authors:** Elvin Aliyev, Thomas Emmler, Jelena Lillepaerg, Sergey Shishatskiy, Nadir Dizge, Volkan Filiz

**Affiliations:** † Institute of Membrane Research, Helmholtz-Zentrum Hereon, Max-Planck Str. 1, 21502 Geesthacht, Germany; ‡ Department of Environmental Engineering, Mersin University, 33343 Mersin, Turkey

## Abstract

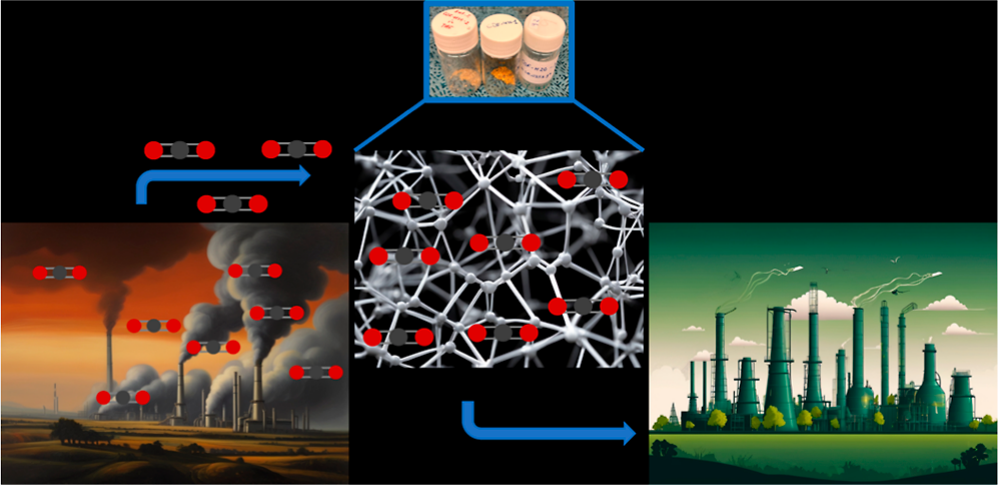

This paper illustrates the gas adsorption properties of newly synthesized nanoporous cross-linked polymer networks (CPNs). All synthesized CPNs possess N-rich functional groups and are used for the utilization of carbon dioxide and methane. Good gas adsorption and selectivities are obtained for all of the samples. Among the materials, HEREON2 outperforms better selectivity for methane separation from nitrogen rather than zeolites, activated carbons, molecular sieves, covalent organic frameworks, and metal–organic frameworks (MOFs). The accessibility of the N-rich functionalities makes these materials potential candidates for the separation of hydrocarbons *via* increased polarizabilities. High-pressure adsorption experiments showed that the synthesized two-dimensional nanoporous materials also have a high affinity toward carbon dioxide. HEREON2 powders showed an increased experimental CO_2_/N_2_ selectivity of ∼25,000 at 50 bar due to the presence of nitrogen groups in the structure. Fourier-transform infrared spectroscopy (FTIR), solid-state NMR, X-ray diffraction, thermogravimetric analysis, energy-dispersive X-ray spectroscopy (EDX), transmission electron microscopy (TEM), and scanning electron microscopy (SEM) were applied for the characterization of the synthesized nanoporous CPNs. The results show a potential new pathway for future CPN membrane development.

## Introduction

Today’s world economy is highly dependent on the oil and gas industry. The use of fossil fuels increases the level of greenhouse gases, such as CO_2_ and CH_4_, in the atmosphere, which drives global warming. In 2015, the Paris Agreement set a target of a maximum increase in temperature of 2 °C, which can only be achieved through the reduction of emissions of these gases by approximately 50% by 2030.^1^ One of the proposed effective methods to tackle this issue is CO_2_ capture and sequestration.^2^ Efficient gas adsorbent materials are highly desired for employment in this mission. The most highlighted technology for industrial CO_2_ capture is the liquid amine scrubbing method due to the reversible reaction between CO_2_ and amine that provides highly effective and selective sorption of the target gas.^3−5^ However, this technique requires a high amount of capital to take action against equipment corrosion and degradation and an intensive energy-demanded regeneration process. In order to make the adsorption technique more cost-effective, several groups around the world are working on the preparation of novel adsorbent materials. In particular, porous solid adsorbents are of interest. The main challenge in this field is that all adsorbents must be evaluated based on their moisture stability, adsorption kinetics, regeneration energy consumption, and cost.^6^ In addition, the adsorbents must possess high adsorption capacities (>2–3 mmol g^–1^) at as low as 0.39% CO_2_ concentrations at adsorption temperatures above 40 °C.^7−10^ Amine-functionalized mesoporous silica,^11−13^ metal–organic frameworks (MOFs),^14−16^ and covalent organic frameworks (COFs)^17,18^ showed a high affinity toward carbon dioxide. Although MOFs are exciting materials for gas adsorption, the presence of a small amount of moisture in the medium slowly degrades their structure.^19−21^ Thus, moisture-stable MOFs are highly recommended in pressure-swing adsorption processes.^22,23^ Unlike MOFs, COFs show chemical stability due to rigid chemical bonding in the structure, which increases their stability. This advantage allows COFs to be employed for CO_2_ capture and storage.^24,25^ For example, it was reported that COF-JLU2 displays remarkable carbon dioxide (217 mg g^–1^) and methane (38 mg g^–1^) uptakes, as well as high CO_2_/N_2_ (77) adsorption selectivity at 273 K.^26^ The strong π–π stacking interactions of the pyrene units in ILCOF-1 enhance the volumetric gas adsorption capacities for carbon dioxide (∼300 L L^–1^) and methane (∼130 L L^–1^) at 298 K and 35 bar.^27^ CO_2_ can further be enhanced by the incorporation of nitrogen-containing groups with increased CO_2_ affinity into the COF structure. The N-rich COFs named COF-SDU1, COF-SDU2, and COF-SDU3 exhibit large CO_2_ uptake of 741, 484, and 331 mg g^–1^, respectively, at 298 K and 45 bar.^28^ However, a significant increase in carbon dioxide uptake at room temperature remains an important challenge for COFs.

Highly cross-linked polymeric networks [cross-linked polymer networks (CPNs)] have gotten much attention due to their high surface area, easy synthetic procedure, low skeleton density, good physicochemical stability, and adsorbing properties of carbon dioxide at ambient temperature^29−31^ and could be useful for gas separation alongside the hyper-cross-linked polymers (HCPs). As a subgroup of highly porous organic polymers, HCPs are one of the most suitable materials as porous media for gas adsorbents due to their excellent stability, low density, and high surface area.^32,33^ Nitrogen-rich covalent triazine framework-based hyper-cross-linked conjugated polymers showed 8.02 wt % CO_2_ storage.^34^ Zhou *et al.* reported that synthesized sulfonated HCPs of intrinsic microporosity (PIMs) showed 298 mg g^–1^ of CO_2_ uptake with a selectivity of 17.9 over N_2_, while aminated PIMs demonstrated high selectivities.^35^ Moreover, microporous polymer networks demonstrated higher CO_2_ uptakes and CO_2_/N_2_ selectivities, which were better than polyimides.^36^ Ionic hyper-cross-linked polymers exhibited selective acid gas adsorption over CH_4_, CO_2_, and N_2_.^37^

Here, we report the synthesis and gas adsorption properties of novel CPNs possessing various nitrogen-containing functional groups (imine, amine, and isocyanurate), as the nitrogen group-bearing compounds are promising materials for CO_2_ adsorption. Although these novel adsorbents show relatively low surface area and pore volume, they have excellent adsorption capabilities and single gas selectivities for CO_2_ and CH_4_ over N_2_, verifying the concept of utilizing attractive interactions of chemical functional groups toward certain gas molecules in designing CPNs. Moreover, we investigate how various structural features of the N-containing functional group present in the structure influence the gas adsorption behavior. Our work shows potential for further application of these N-containing functional groups in the preparation of 2D CPN membranes, which can be an advantage over HCPs as the latter ones are rigid materials and cannot be transformed into solution-cast membranes.

## Experimental Section

### Materials

3,3′,5,5′-Tetramethylbenzidine (TMB, 98%), 1,3,5-benzenetricarbonyl chloride (TMC, 98+%), benzimidazole (BI, 99%), and glacial acetic acid (CH_3_CH_2_COOH, 99+%) were purchased from Alfa Aesar (Heysham, UK). Tris(3-hydroxypropyltriazolylmethyl)amine (THPTAMA, 95%), triethylamine (TEA, Et_3_N, 99%), 1,3,5-tris(2-hydroxyethyl)isocyanurate (THEIC, 97%), benzene-1,3,5-tricarboxaldehyde (97%), 1,3,5-trimethylbenzene (mesitylene, 98%), *N*,*N*-dimethylformamide (DMF, anhydrous, 99.8%), tetrahydrofuran (THF, anhydrous, 99.8%), and 1,4-dioxane (anhydrous, 99.8%) were purchased from Sigma-Aldrich (St. Louis, Missouri, USA). All chemicals were of reagent grade and used as purchased.

### Synthesis of HEREON1

For the synthesis of HEREON1, *ca.* 0.05 g of benzene-1,3,5-tricarboxaldehyde (BTA, ∼0.3 mmol) and ∼0.11 g of 3,3′,5,5′-tetramethylbenzidine (TMB, ∼0.45 mmol) were dissolved in 5 mL of mesitylene and 5 mL of 1,4-dioxane, respectively, in two different 20 mL sealed flasks. In the next steps, the BTA solution was added into a 25 mL round-bottom flask, followed by 1 mL of glacial acetic acid and the TMB solution additions. After these steps, the flask was mounted to a reflux condenser, and the reaction started at 60 °C. The obtained product in the form of yellow powder was vacuum filtered and sequentially washed with methanol, THF, and water, and the resulting material was vacuum-dried (Scheme S1).

### Synthesis of HEREON2

The synthesis of HEREON2 was conducted in a 25 mL round-bottom flask through the reaction of 10 mL of 1,3,5-benzenetricarbonyl chloride (TMC, ∼0.05 g, ∼0.2 mmol) solution in mesitylene and 10 mL of tris(3-hydroxypropyltriazolylmethyl)amine (THPTAMA, ∼0.08 g, ∼0.2 mmol) solution in DMF in the presence of TEA (1 mL) at 70 °C for 24 h. The obtained red COF particles were vacuum filtered and sequentially washed with methanol, THF, and water, and the resulting material was vacuum-dried (Scheme S2).

### Synthesis of HEREON3

HEREON3 synthesis was conducted with the reported protocol for HEREON2. However, the 1,3,5-benzenetricarbonyl chloride (TMC, ∼0.1 g, ∼0.4 mmol) solution (10 mL) was reacted with the 1,3,5-tris(2-hydroxyethyl)isocyanurate (THEIC, ∼0.1 g, ∼0.4 mmol) solution (10 mL). The synthesized greenish-white powder was vacuum-filtered and sequentially washed with methanol, THF, and water, and the resulting material was vacuum-dried (Scheme S3).

### Characterization

Fourier transform infrared (FTIR) spectra were recorded in attenuated total reflectance mode on a Bruker ALPHA FT-IR spectrometer (Bruker, Ettlingen, Germany). The transmittance measurements were collected at ambient temperature in a spectral range of 400–4000 cm^–1^ at a resolution of 4 cm^–1^ with 64 scans. Solid-state NMR (SSNMR) experiments were performed on a Bruker Avance II 400 spectrometer (Bruker, Rheinstetten, Germany) equipped with a 4 mm double resonance probe. ^13^C{^1^H} cross-polarization (CP) magic angle spinning (MAS) spectra were acquired using ramped polarization transfer with a ^1^H 90° pulse length of 4.0 μs, contact time of 1 ms, and a repetition delay of 4 s. Two-pulse phase-modulated decoupling was used during the acquisition. All experiments were conducted at a spinning frequency of 13 kHz at room temperature. A D8 discover X-ray diffractometer (Bruker, Ettlingen, Germany) with Cu Kα radiation (λ = 1.54184 Å, 50 kV, 1000 mA) was applied for the X-ray diffraction (XRD) experiments of the CPN samples at a scanning rate of 1° min^–1^. Airtight sample holders (Bruker, Ettlingen, Germany) were used to prevent any contamination of the samples. Thermogravimetric analysis (TGA) was used to investigate the mass loss of the synthesized CPN samples as a function of the temperature. The analysis was carried out on the Netzsch TG209 F1 Iris instrument (Netzsch, Selb, Germany) under argon flow (50 mL min^–1^) in the temperature range from 25 to 800 °C at 5 K min^–1^. Sample porosity and BET area were determined by the N_2_-adsorption on a Micrometrics ASAP2020MPHD analyzer [Micromeritics Instrumental Corp., Norcross (GA), USA]. N_2_-isotherms were measured at 77 K. The surface area was calculated by the BET theory between 0.05 and 0.3 *p*/*p*_0_. During the data fitting, it was made sure that no negative BET constant occured. The apparent micropore distributions were calculated in the MicroActive Software [Micromeritics Instrumental Corp., Norcross (GA), USA] from N_2_ adsorption data employing the DFT pore size method, assuming a cylinder-pore geometry and the “N_2_-Cylindrical Pores–Oxide Surface” model. Secondary electron images and energy-dispersive X-ray (EDX) spectra were taken at accelerating voltages of 2–3 kV. For TEM analysis, CPN samples were dispersed in THF in order to prepare a 0.1 wt % concentration and sonicated for 30 min. 2 μL of each dispersion was drop cast onto a carbon film grid (S160, Cu 200 mesh) and analyzed on a Tecnai G^2^ T20 (FEI Company, USA, Hillsboro). The gas adsorption analysis of the CPN particles was conducted on a magnetic suspension balance (MSB, Rubotherm Series IsoSORP sorption analyzer, TA Instruments, New Castle, DE, USA) with an uncertainty of 10 μg. The sample of CPNs in powder form was evacuated at 120 °C for 24 h in order to remove the gases adsorbed from the atmosphere. The adsorption measurements were conducted stepwise from a high vacuum to 50 bar for the CH_4_, N_2_, O_2_, and CO_2_ series. Pressure sensors with an accuracy of ±0.5% of full scale were used. The specific uptake was analyzed, considering gas buoyancy.^38^ The uncertainty of this measurement was estimated according to a method described elsewhere.^39^ The density of the sample was estimated *in situ* with helium at 30 °C in the pressure range of 10^–5^ to 50 bar.

The equilibrium concentration *C* of gas in the polymer for a given gas pressure *p* was correlated using an adsorption isotherm-generalized Langmuir type
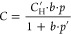
1where *C*_H_^′^ is the Langmuir sorption capacity related to addition sorption owing to the nonequilibrium volume, and *b* is the Langmuir affinity parameter.

The dual sorption model was applied as well in the form
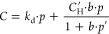
2where *k*_d_ is Henry’s law constant, and *p* is the feed pressure. Isotherms at high pressure are presented in terms of fugacity.

## Results and Discussion

In our research, three types of CPNs were synthesized through the polycondensation reaction. The new CPNs named HEREON1, HEREON2, and HEREON3 are described in the Supporting Information. The reaction schemes (Schemes S1–S3) show that the new CPNs are imine- and ester-based. HEREON1 synthesis is carried out by the polycondensation reaction between benzene-1,3,5-tricarboxaldehyde and 3,3′,5,5′-tetramethylbenzidine with the elimination of two water molecules. The synthesis of HEREON2 and HEREON3 proceeds with the elimination of HCl during the reactions of trimesoyl chloride with tris(3-hydropropyltriazolylmethyl)amine and 1,3,5-tris(2-hyroxyethyl)isocyanurate, respectively. In the cases of HEREON1 and HEREON3, 12-membered imine and six-membered ester rings are formed by the dehydration and dehydrochlorination reactions, while in the case of HEREON2, the HCl elimination causes the formation of nonuniform rings. The rationale behind choosing these monomers was to synthesize nitrogen-rich porous structures that have affinities toward CO_2_ and could be used as adsorbents in the gas industry. However, flexible sites in the selected monomers lead to the collapse of the ordered porous structure, resulting in low adsorption properties.

**Figure 1:**
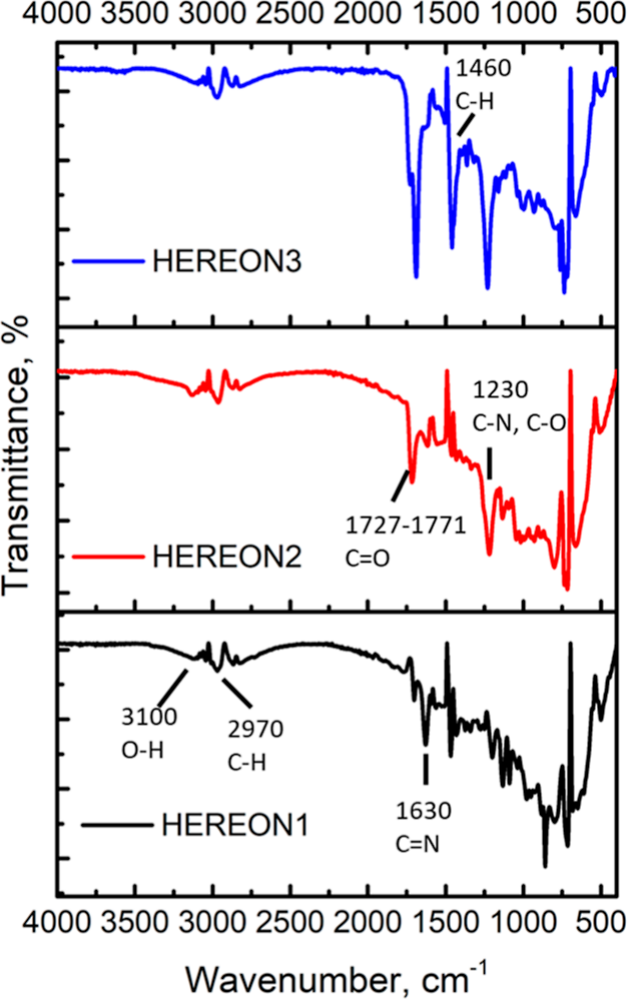
FTIR results of the CPN samples.

FTIR spectroscopy supports the proposed structures of the synthesized CPNs (Figure 1). In all samples, several characteristic vibration peaks are observed with significantly different intensities. C–H stretching vibrations of the alkane groups are visible at 2970 cm^–1^, while intermolecularly bonded O–H vibrations can be found at 3100 cm^–1^. These vibrations in HEREON2 and HEREON3 are more intense than the vibrations of HEREON1. This is related to the unreacted –OH groups in both structures. The −C=O stretching vibrations of the edge carbonyl groups are between 1727 and 1771 cm^–1^. The C–H stretching vibrations of the methylene and methyl groups are observed at 1460 cm^–1^. The peak at ∼1230 cm^–1^ shows the presence of C–N and C–O stretching vibrations of the aromatic amine and ester linkages in the structures, respectively. For the HEREON1 sample, the peak corresponding to the −C=N stretching vibrations is seen at 1630 cm^–1^, confirming the formation of an imine linkage. In addition, the C–N stretching vibrations of aromatic amines are observed with significantly reduced intensity, indicating that a small amount of the 3,3′,5,5′-tetramethylbenzidine moieties that have not formed a six-membered ring is also present in the HEREON1 sample. For HEREON2 and HEREON3, strong peak intensities of the C–N and C–O stretching vibrations imply the formation of alkyl aryl ether linkages. To verify the structures of the CPNs, ^13^C CP MAS NMR analysis was conducted, and the results with peak assignments are shown in Figure S1.

The results suggest that all of the reactions were successful, confirming the proposed structures for the CPN samples. For HEREON1, the framework structure consisting of benzene and benzidine rings is represented by the aromatic carbon signals at 127 and 138 ppm and by a strong peak at 18 ppm resulting from the methyl groups (−CH_3_) of the benzidine moieties. The peak at 150 ppm can be ascribed to the aromatic amine moieties, and the imine moieties are seen at ∼160 ppm, verifying the condensation reaction product.^40^ At ∼190 ppm, the edge carbonyl groups are also detected for HEREON1 (Figure S1a). For HEREON2, signals of THPTAMA units are shown at 30–63 ppm for aliphatic carbons and 126 and 145 ppm for the triazole moieties. The benzene rings resulting from trimesoyl chloride moieties are revealed at 131 ppm. A strong signal at 165 ppm is related to the −COO groups, confirming the condensation reaction. A small peak at 169 ppm belongs to the unreacted −COCl groups situated at the edges of the two-dimensional structure. For HEREON3, ^13^C NMR signals of THEIC units are visible at 42, 62, and 150 ppm, as well as the signal of trimesoyl chloride moieties at 131 ppm. Again, the signals of the −COO groups and −COCl groups at the edge are observed, indicating the condensation reaction.

**Figure 2:**
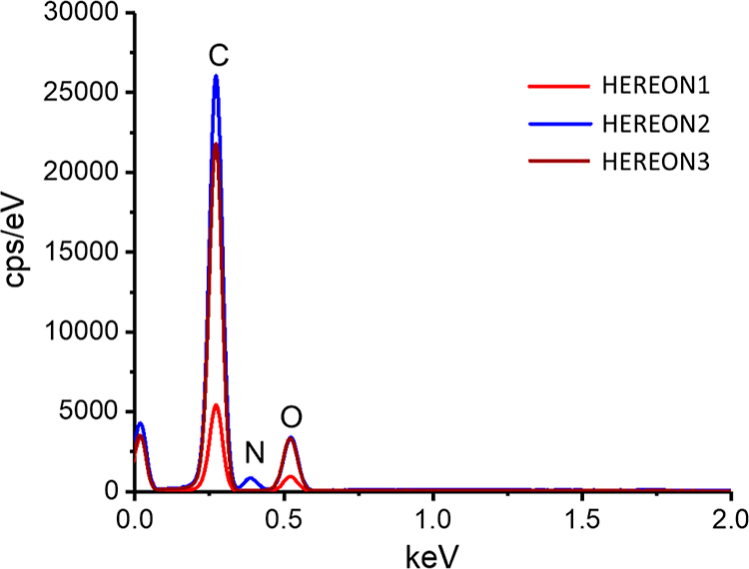
EDX results of the synthesized CPN particles.

The EDX investigation of CPNs supports the results of FTIR and SSNMR. Figure 2 shows that all the atoms are distributed through the structure of the synthesized particles. Nitrogen was undetectable for HEREON1 and HEREON3. The reasons for this could be explained by (a) nitrogen producing a very weak response and (b) nitrogen having only a K_α_ shell in the covalent bonding with the carbon atoms and its electrons being shared. However, in HEREON2, one nitrogen atom has not covalently bonded to the carbon atoms in the structure, and for this reason, it was possible to detect its presence in the structure.

Initially it was thought that the synthesized materials were COFs instead of CPNs. COFs are crystalline powders in general, meaning that such materials must show strong powder XRD peaks. However, it has been reported that during COF synthesis, “crystallization problems” can occur.^41^

**Figure 3:**
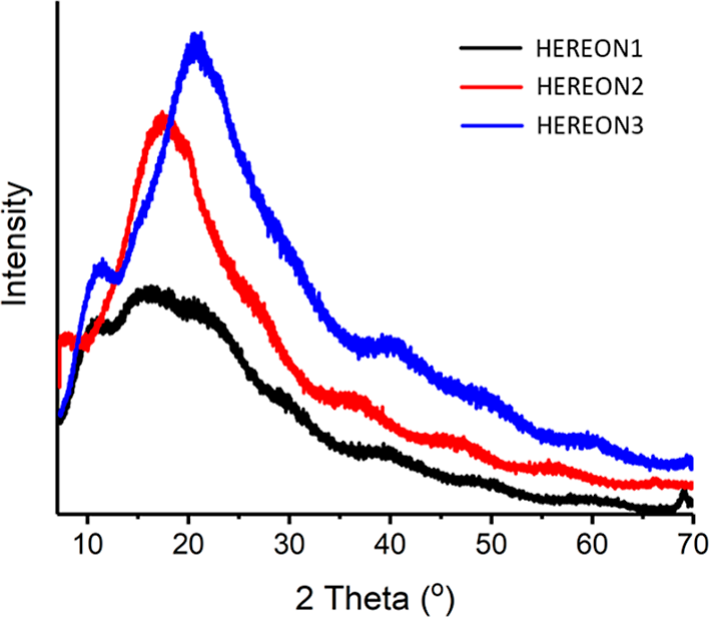
Experimental PXRD results for the CPN samples.

As shown in Figure 3, PXRD experiments revealed that the synthesized CPN particles do not show high crystallinity, as was reported for the TpBD COF samples.^42^ The reason is that the building blocks are flexible, and the formed structural pores collapsed, making these materials amorphous. However, the samples show some PXRD reflections, leading us to claim that the CPNs are amorphous, indicating less structural order, which was also reported for superacid catalyzed low-temperature triazine-based frameworks.^43^

To confirm the possible crystallinity of the CPN transmission electron microscope (TEM), experiments were conducted on the samples (Figure S2).

HEREON1 did not show any crystalline planes; however, it was revealed that this sample is layered. The crystallinity disorder and the broadening of the reflections are most likely due to the deviation from the ideal stacking of the two-dimensional layers governed by the electrostatic and attractive dispersion forces.^27,44,45^ Furthermore, such a kind of staggered stacking can reduce the pore volume and, thus, limit the uptake of high-molecular-weight compounds.

HEREON2 showed a network-like construction containing a porous structure. The same structure was seen in Pd-anchored COF-TB COF.^46^ Thus, crystalline planes were not observed.

Therefore, rectangular needle-like structures were seen in HEREON3. This could be explained by the formation of molecular crystals that give a sharp peak in the PXRD results. However, the peak broadens, and the continuous sharp peaks that are seen in crystalline frameworks were not observed.

Based on PXRD and TEM analysis, it was concluded that the synthesized new materials have amorphous features, even though some crystalline planes were detected in the structures.

Figure S3 shows the scanning electron microscope (SEM) images of the synthesized CPNs. All compounds exhibit agglomerates of particles with irregular, roundish shapes that support the results of TEM experiments.

**Figure 4:**
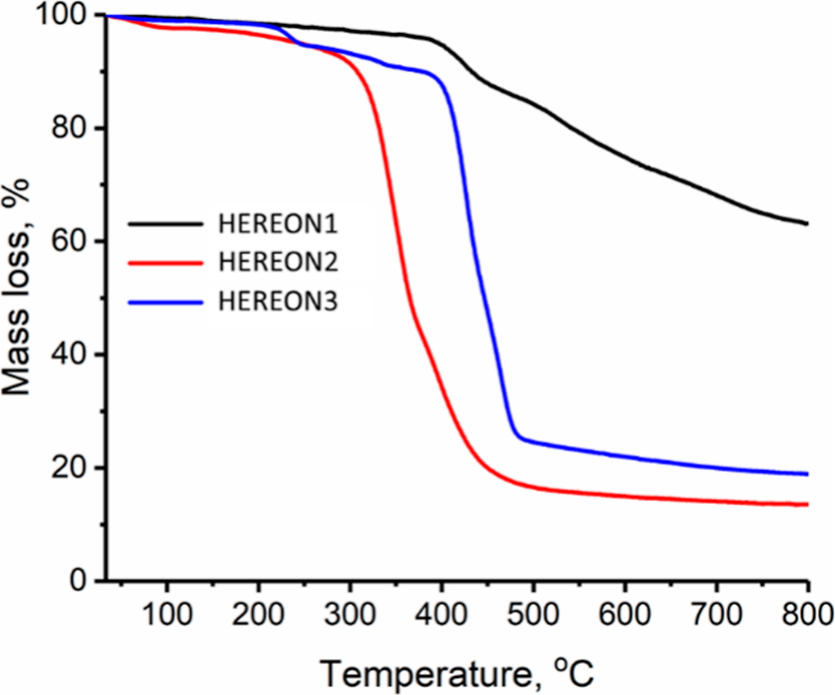
Thermogravimetric patterns of the CPN samples between 25 and 800 °C under an argon flow.

Thermogravimetric analysis showed that the synthesized CPNs are stable up to 400 °C in the case of HEREON1, while the thermal stabilities of HEREON2 and HEREON3 are slightly lower (Figure 4).

In the case of HEREON1, at 400 °C, the first degradation step starts, which can be attributed to the decomposition of edge carbonyl and amine groups from the structure with a mass loss of ∼10%. This is followed by a gradual mass loss of ∼28% up to 800 °C, corresponding to the degradation of the six-membered rings. The residual mass at the end of the experiment is ∼62%. The sample HEREON2 shows a TGA curve comparable to HEREON1. A small mass loss up to 100 °C could be explained by the evaporation of the residual water molecules trapped in the pores of the network-like structure during sample transfer to the TGA apparatus. A sharp degradation step between 250 and 550 °C is explained by the degradation of the structure, suggesting the elimination of CO_2_. Up to 800 °C, approximately 90% of the structure is degraded. The degradation of HEREON3 is accompanied by the decomposition of the unreacted –OH and −COCl groups between 200 and 360 °C, with a mass loss of ∼10%. A steep decrease in the mass loss between 360 and 750 °C suggests the structural destruction of the sample. The different TGA profiles suggest that the structure of HEREON1 is much more stable than that of HEREON2 and HEREON3. The stiff covalent bonds of HEREON1 prevent the thermochemical reactions in the molecule, and the residual ash mass possesses mainly porous nitrogen-containing carbon. The flexible linkages in HEREON2 are the weak sides for decomposition, and this compound starts to decompose above 200 °C. Based on the thermal degradation of HEREON3, it can be judged that ester linkages are more prone to degradation, even though the structure can be stable after synthesis.

**Figure 5:**
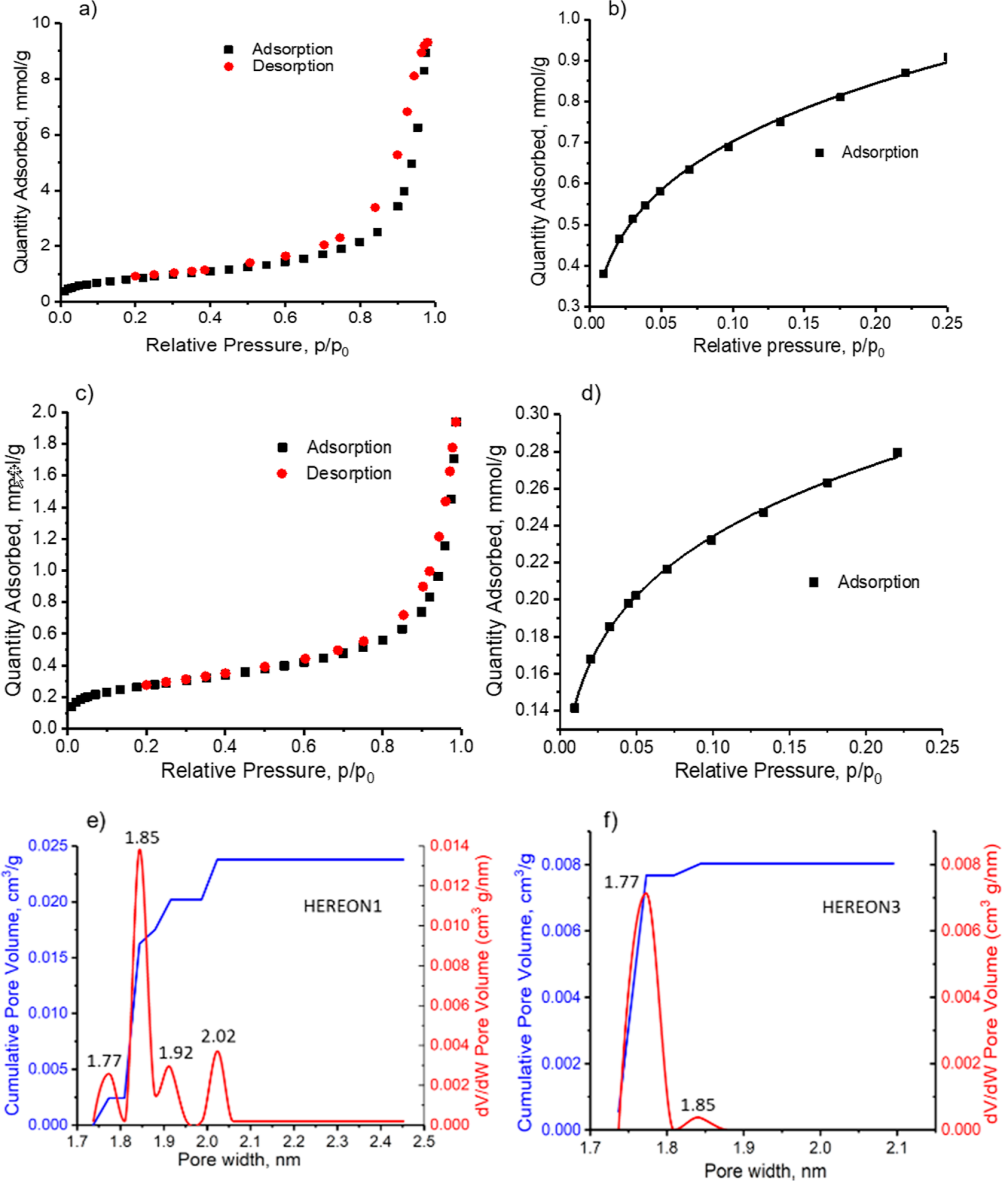
Nitrogen adsorption–desorption isotherms of HEREON1 (a,b) and HEREON3 (c,d). Pore size distribution of HEREON1 (e) and HEREON3 (f).

For the evaluation of the existence of possible porosities and the specific surface areas of synthesized HEREON1 and HEREON3, nitrogen adsorption–desorption experiments were conducted at 77 K. Before the experiments, the samples were degassed at 120 °C for 24 h. As shown in Figure 5a–d, the adsorption isotherms for the CPNs are a combination of type Ib and type II, according to the IUPAC classification.^47^

Reversible isotherms given in Figure 5a–d show that the synthesized CPNs possess a microporous nature due to interparticle aggregation. The isotherms demonstrate that the synthesized materials have relatively small external surfaces. The type Ib characteristic, *i.e.*, a steep uptake at low *p*/*p*_0_ (Figure 5b,d) indicates that the synthesized CPNs possess microporosity, ranging between 1.77 and 2 nm (Figure 5e,f). In the case of HEREON1, the main pore size is 1.85 nm, with distributions in pore sizes such as 1.77, 1.92, and 2.0 nm. HEREON3 displays a more uniform pore size distribution, where the majority of micropores are 1.77 nm in diameter and micropores of *d* = 1.85 nm are present in a minor amount. The presence of multiple pore sizes can be attributed to the topology and flexibility of the ligands used in the synthesis. This factor, in our opinion, affects the uniformity of the nanopores. The second pore size can be attributed to the staggered packing of the 2D layers of the CPNs, which is the result of the electrostatic dispersion force. The Brunauer–Emmett–Teller (BET) and Langmuir surface areas of the samples are SA_BET_ = 70 m^2^ g^–1^ and *S*_Lang_ = 85 m^2^ g^–1^ for HEREON1, and SA_BET_ = 21 m^2^ g^–1^ and *S*_Lang_ = 27 m^2^ g^–1^ for HEREON3, respectively. The BET surface areas obtained are considerably lower than the previously reported COFs such as COF-42 (710 m^2^ g^–1^), COF-43 (620 m^2^ g^–1^),^48^ COF-1 (711 m^2^ g^–1^),^49^ COF-LZU1 (410 m^2^ g^–1^),^50^ and (Et)_25_-H_2_P-COF (1326 m^2^ g^–1^).^51^ However, values for materials under study are higher than the amorphous TAPB-PDA COF (18 m^2^ g^–1^),^52^ and graphite (10 m^2^ g^–1^)^53^ or close to some of the reported amine-functionalized microporous organic polymers (72 m^2^ g^–1^).^54^ The total pore volume calculated at *p*/p_0_ = 0.99 is 0.33 and 0.07 cm^3^ g^–1^ for HEREON1 and HEREON3, respectively, which is lower than that of COF-SDU3 (0.53 cm^3^ g^–1^),^28^ ILCOF-1 (1.21 cm^3^ g^–1^),^27^ COF-JLU2 (0.56 cm^3^ g^–1^),^26^ COF-42 (0.31 m^3^ g^–1^),^48^ COF-43 (0.36 m^3^ g^–1^),^48^ COF-1 (0.30 m^3^ g^–1^),^55^ and COF-6 (0.32 m^3^ g^–1^).^55^ This could be explained by the phenomenon that the nitrogen molecules could not enter the pores of the networks, whereas the 2D layers are in the staggered form. The lower surface area could be explained by the presence of the side functional groups that cover the inner and outer surfaces of the CPN samples. However, the diffusion of nitrogen molecules between the 2D layers is possible. Chandra *et al.*(42) reported that the introduction of a functional group at the 3,3′ position in the biphenyl ring system disturbs the planarity of the diamine ligands. The diamine ligands of HEREON1 contain methyl functionalization at the 3,3′ and 5,5′ positions, which means the planarity of the diamine groups is disturbed. This can be a reason for the low crystallinity and, consequently, the lower surface area. Considering this factor, we claim that the presence of different functional groups in the ligands used for the synthesis of HEREON2 and HEREON3 affects the crystallinity and packing of the two-dimensional nanolayers.

**Figure 6:**
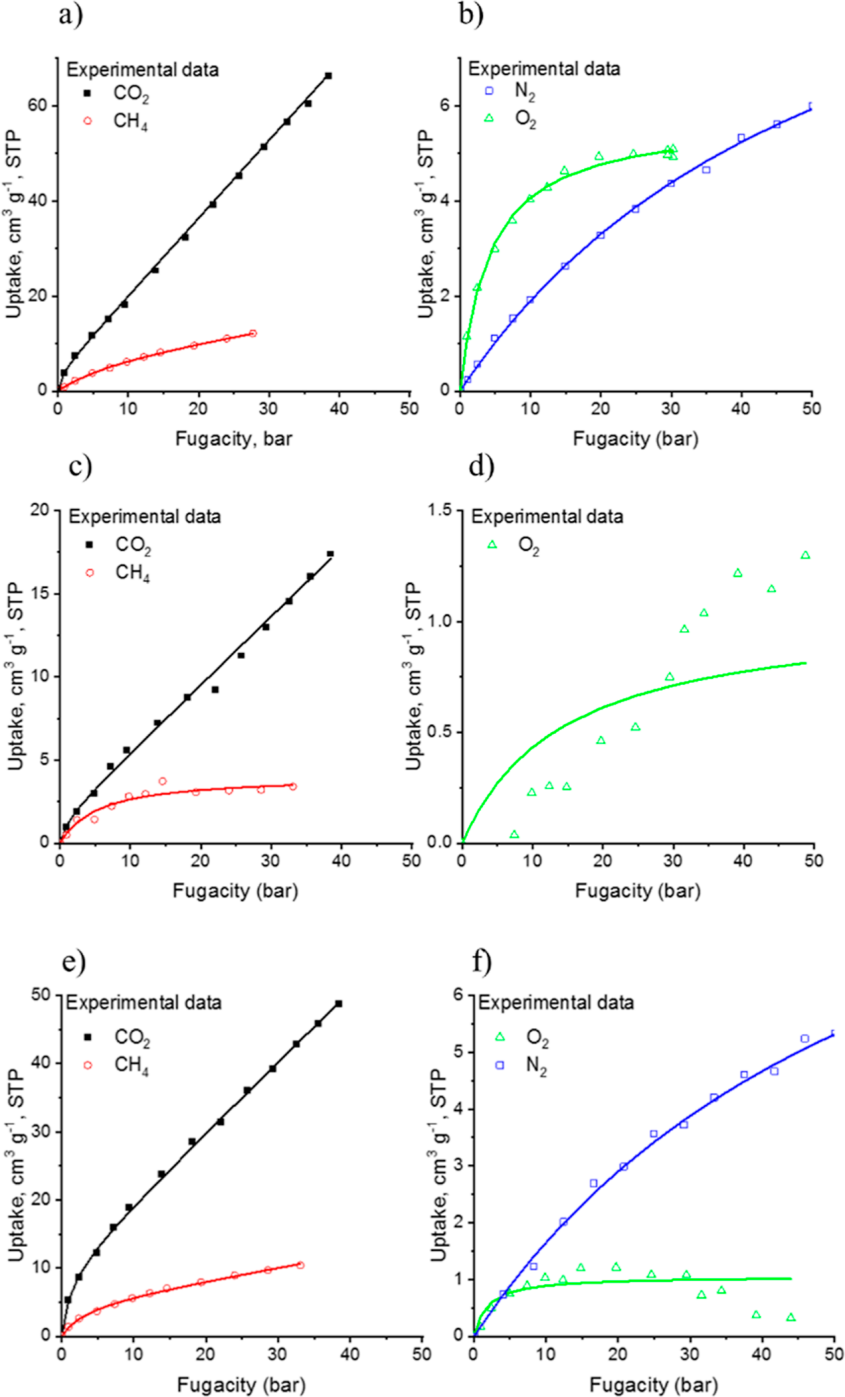
Experimental adsorption isotherms of CO_2_, CH_4_, O_2_, and N_2_ on (a,b) HEREON1, (c,d) HEREON2, and (e,f) HEREON3. Solid lines represent the Langmuir model (eq 1) and dual sorption model (eq 2) isotherms. Uncertainties of data are the size of the graphical symbols.

During the conceptualization of the work, it was planned to prepare CPNs that have an affinity toward CO_2_. Since synthesized CPNs (HEREON1, HEREON2, and HEREON3) contain a variety of functional groups distributed through the frameworks, adsorption experiments for CH_4_, N_2_, O_2_, and CO_2_ gases at 303 K were performed. The idea was to understand the behavior of the CPNs toward the gas molecules and to lay the basis for the development of membranes from these CPNs for the separation of CO_2_ in the industry at relevant pressures. In Figure 6, the experimental data and the Langmuir model adsorption isotherms of the CPNs are shown.

Although the BET surface areas of the synthesized CPNs are low, they show high gas uptake. For the CO_2_ isotherm, it can be found that the synthesized CPNs are indeed CO_2_-philic, as expected. Thus, all of the uptake curves can fit the Langmuir model described with eq 1.

The isotherm shows that with the increase of pressure the uptake of CO_2_ increases and reaches 67 cm^3^ g^–1^ for HEREON1, 17 cm^3^ g^–1^ for HEREON2, and 49 cm^3^ g^–1^ for HEREON3 samples. Among the synthesized CPNs, HEREON1 exceeds the CO_2_ uptake values of SNW-1 (50 cm^3^ g^–1^).^56^ Parallel experiments with CH_4_, O_2_, and N_2_ were conducted as well. HEREON1 exhibited 5.9 cm^3^ g^–1^ uptake for N_2_, 5.32 cm^3^ g^–1^ uptake for O_2_, and 17 cm^3^ g^–1^ uptake for CH_4_. Interestingly, nitrogen and oxygen adsorptions of HEREON2 and HEREON3 were very low and almost undetectable. The reason for such a lack of adsorption ability could be the polarizability and quadrupole moments of these molecules. HEREON2 does not adsorb nitrogen at all, while HEREON3 adsorbs O_2_ at 10 bar with an uptake value of 1.0 cm^3^ g^–1^ (with the increase of the pressure above 30 bar, the adsorption value decreases). During experiments, the HEREON2 adsorption isotherm for O_2_ was barely positive, while the adsorption isotherm for N_2_ was always negative after filtering the major negative adsorption points, which means that such kinds of materials do not have an affinity toward nitrogen. Employing the Langmuir model (eq 1), it was predicted that HEREON2 must adsorb 0.17 cm^3^ g^–1^, while the maximum nitrogen uptake value predicted for HEREON3 is 5.3 cm^3^ g^–1^ (Figure 6f). These predictions were not supported by the experimental values. The adsorption of methane was higher than that of nitrogen and reached 3.52 and 11.0 cm^3^ g^–1^ at 35 bar in the case of HEREON2 and HEREON3, respectively. In Table 1, the CO_2_ capture results of the synthesized materials, along with literature data, are shown.

**Table 1:** CO_2_ Sorption Results for the Synthesized CPNs along with Literature Data for COFs and Crosslinked Polymers

samples	CO_2_ capacities, cm^3^ g^–1^	CO_2_/N_2_	CO_2_/CH_4_	refs
SNW-1	50	10	5	(56)
CTF-1	55^a^	18^b^		(57)
TpPa-COF (MW)	111^a^	32^c^		(58)
COF-JLU2	110^a^	77^c^	5.7^c^	(26)
ACOF-1	90^a^	40^c^	15.4^c^	(59)
PIM-Trip-NH_2_	67	26^e^	8^f^	(35)
HCP-3	>90	42.9^e^		(60)
C3HCP-3	>50	35.53		(61)
mPAF-1/16	134	5.5^d^	2^d^	(62)
HEREON1	67	11.3^d^	4.0^d^	this work
HEREON2	17	>100^d^	4.7^d^	
HEREON3	49	9.3^d^	3.8^d^	

a Low-pressure CO_2_ capacity was measured at 273 K at 1 bar.

b Determined from the experiment breakthrough with CO_2_/N_2_ (10:90 v/v) at 298 K and 1 bar.

c Determined from Henry’s law.

d Determined from the Langmuir model.

e IAST CO_2_/N_2_ selectivity (15:85 v/v) at 298 K and 1 bar.

f IAST CO_2_/CH_4_ selectivity (50:50 v/v) at 298 K and 1 bar.

Table 1 shows that the newly synthesized HEREON2 shows high CO_2_/N_2_ selectivities, exceeding the results reported for COF-JLU2, (COOH)_100_-H_2_P-COF,^51^ FCTF-1,^57^ and HCPs.^63^ However, due to the low polarizability of nitrogen, the adsorption in the HEREON2 framework decreases drastically, while CO_2_ and CH_4_ adsorption increase, reaching high sorption selectivities for CO_2_ and CH_4_ over N_2_ of ∼25,000 and ∼5820 at 50 bar, respectively. These selectivities were solely calculated based on experimental values at 50 bar.

**Figure 7:**
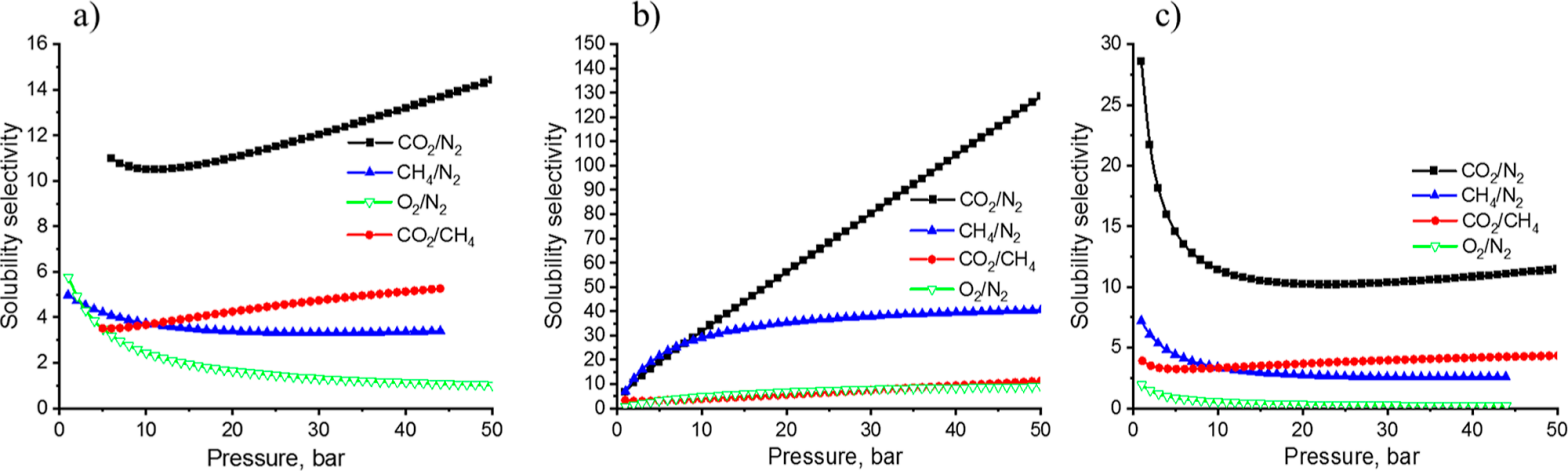
Solubility selectivities of (a) HEREON1, (b) HEREON2, and (c) HEREON3 *versus* applied pressure.

Figure 7 shows the evolution of the solubility (adsorption) selectivities of binary gas mixtures of CO_2_/N_2_, CH_4_/N_2_, O_2_/N_2_, and CO_2_/CH_4_ as a function of applied pressure. The selectivities were calculated according to the Langmuir sorption model for every single gas. As is described in the model, at 10 bar, the CO_2_/N_2_ selectivity is 10, 30, and 11 for HEREON1, HEREON2, and HEREON3, respectively. With the pressure increase, the selectivity also increases and reaches 14 for HEREON1, 120 for HEREON2, and 11.5 for HEREON3. The CH_4_/N_2_ selectivity is calculated to be 4 at 10 bar, and with further pressure increases, the selectivity decreases slightly for HEREON1 and HEREON3. However, HEREON2 shows a selectivity value of 30 at 10 bar, and with the increase of pressure, this value hovers around 30. The experiments showed that with the increase of pressure, the O_2_/N_2_ selectivity decreased in the cases of HEREON1 and HEREON3. This is explained by the higher adsorption of nitrogen over oxygen at high pressures, which could be the result of surface adsorption of nitrogen by the CPNs. Thus, these polymers show repulsive forces on oxygen, and their adsorption is reduced, which results in low O_2_/N_2_ selectivity. This phenomenon perfectly fits with the results of HCPs reported by Maleki.^64^ This value was barely detectable for HEREON2 due to the absence of interaction between the CPN and oxygen molecules. The higher CO_2_/N_2_ selectivities are related to the significantly higher polarizability (29.1 × 10^–25^ cm^3^) and quadrupole moment (13.4 × 10^–40^ cm^2^) of CO_2_ over those for N_2_ (polarizability: 17.4 × 10^–25^ cm^3^; quadrupole moment: 4.7 × 10^–40^ cm^2^). When it comes to the selectivity of CH_4_ over N_2_, these data are five times lower for HEREON1 and ∼1.5 times lower for HEREON3. The quadrupole moment of CH_4_ is zero; however, the polarizability (25.9 × 10^–25^ cm^3^) is high.^65^ These values confirm that the polarizability of CH_4_ molecules by the synthesized CPNs is in the order of HEREON2 > HEREON3 > HEREON1. It means that the separation of CH_4_ from N_2_ is solely dependent on the adsorbent–adsorbate interaction, where polarizability plays an important role. Thus, the functionalization of organic linkers with molecules able to polarize methane could be a potential approach for highly selective hydrocarbon upgrade processes. Our adsorption-based results are significantly higher than the results reported for carbon molecular sieves (CMSs) (1.9), silicalite pellets (3.4), and activated carbons (3.0–4.0). Molecular simulation studies conducted on several MOFs (BERGAI01, PEQHOK, and GUSLUC) as potential candidates showed moderate selectivities between 7.7 and 8.8 for CH_4_ separation.^66^ Synthesized CPNs show better experimental results than those simulations exceeding those of other reported adsorbents in this field (HEREON1 and HEREON3 are exceptional). Table 2 compares synthesized CPNs and other adsorbents, such as zeolites, activated carbons, molecular sieves, and MOFs reported in the literature.

**Table 2:** CH_4_/N_2_ Selectivities of Different Adsorbents as a Comparative Study with Our Results^a^

adsorbents	*S*_(ads)_, CH_4_/N_2_	condition	method	refs
Linde 5A zeolite	2.20	298 K, 10 bar	a	(67)
SAPO-34 zeolite	3.00	298 K, 10 bar	a	(67)
MFI zeolite	9.50	298 K, 10 bar	b	(68)
LTA zeolite	5.00	298 K, 10 bar	b	(68)
DDR zeolite	10.5	298 K, 10 bar	b	(68)
F30-470 Degussa activated carbon	3.20	303 K, 10 bar	c	(69)
anthracite-based activated carbon	1.73	298 K, 10 bar	c	(70)
Xtrusorb A754 activated carbon	2.11	303 K, 10 bar	c	(71)
Bayer KEL2200 5A molecular sieve	1.90	303 K, 10 bar	c	(69)
Taixi anthracite-based carbon molecular sieve (CMS)	1.50	298 K, 10 bar	c	(72)
Cu-BTC	3.00	298 K, 10 bar	b	(68)
IRMOF-11	4.00	298 K, 10 bar	b	(68)
BERGAI01	8.80	298 K, 10 bar	b	(66)
PEQHOK	8.40	298 K, 10 bar	b	(66)
GUSLUC	7.70	298 K, 10 bar	b	(66)
HEREON1	4.50	303 K, 10 bar	c	this work
HEREON2	30.0	303 K, 10 bar	c	
HEREON3	4.00	303 K, 10 bar	c	

a a—breakthrough experiments (CH_4_/N_2_, 50/50), b—mixed gas simulations (CH_4_/N_2_, 50/50), and c—single gas adsorption experiments.

Table 2 shows that under the same conditions, the CH_4_/N_2_ adsorption selectivity of HEREON2 explicitly exceeds that of zeolites, activated carbons, carbon molecular sieves, and MOFs, making this material a potential candidate for CH_4_ separation from N_2_. The same results for HEREON1 and HEREON3 were either higher than or equal to the values of some of the compared materials. The experiments showed that with the increase of pressure, the O_2_/N_2_ selectivity decreased in the cases of HEREON1 and HEREON3. However, we could calculate the selectivity of the O_2_/N_2_ for HEREON2, and this value is around 5. HEREON3 shows low selectivity (∼3) for oxygen over nitrogen at low pressures. With the increase of pressure, the selectivity decreases below 1. The almost equal polarizabilities of nitrogen (17.4 × 10^–25^ cm^3^) and oxygen (15.8 × 10^–25^ cm^3^)^65^ explain the lower O_2_/N_2_ selectivity. It seems that with the increase of pressure, the polarization of nitrogen by the pores of HEREON3 increases, leading to a 4-fold increase of adsorption capacity (from 0.15 to 0.6 cm^3^ g^–1^), while oxygen adsorption remains the same.

**Figure 8:**
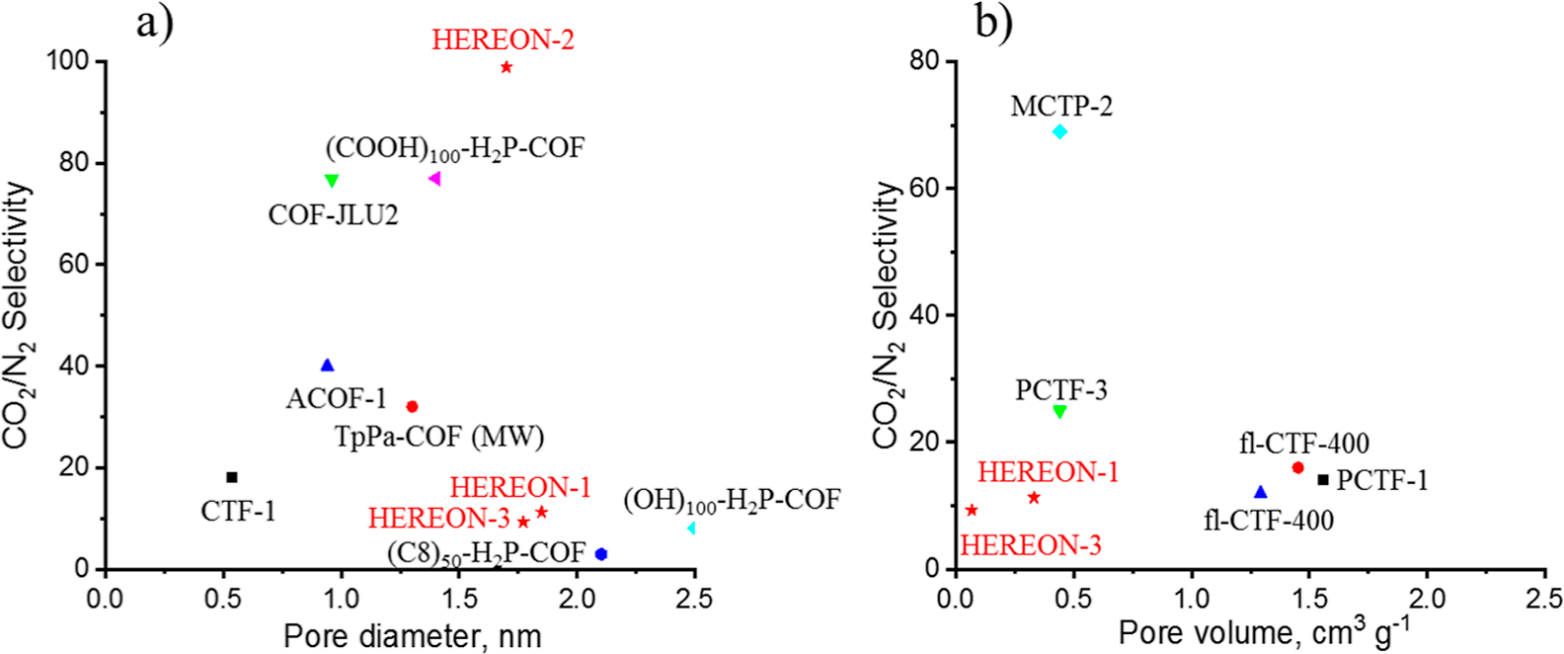
CO_2_/N_2_ selectivity as a function of pore diameter (a) and pore volume (b). For the plots, data from refs (24 and 73) were used.

**Figure 9:**
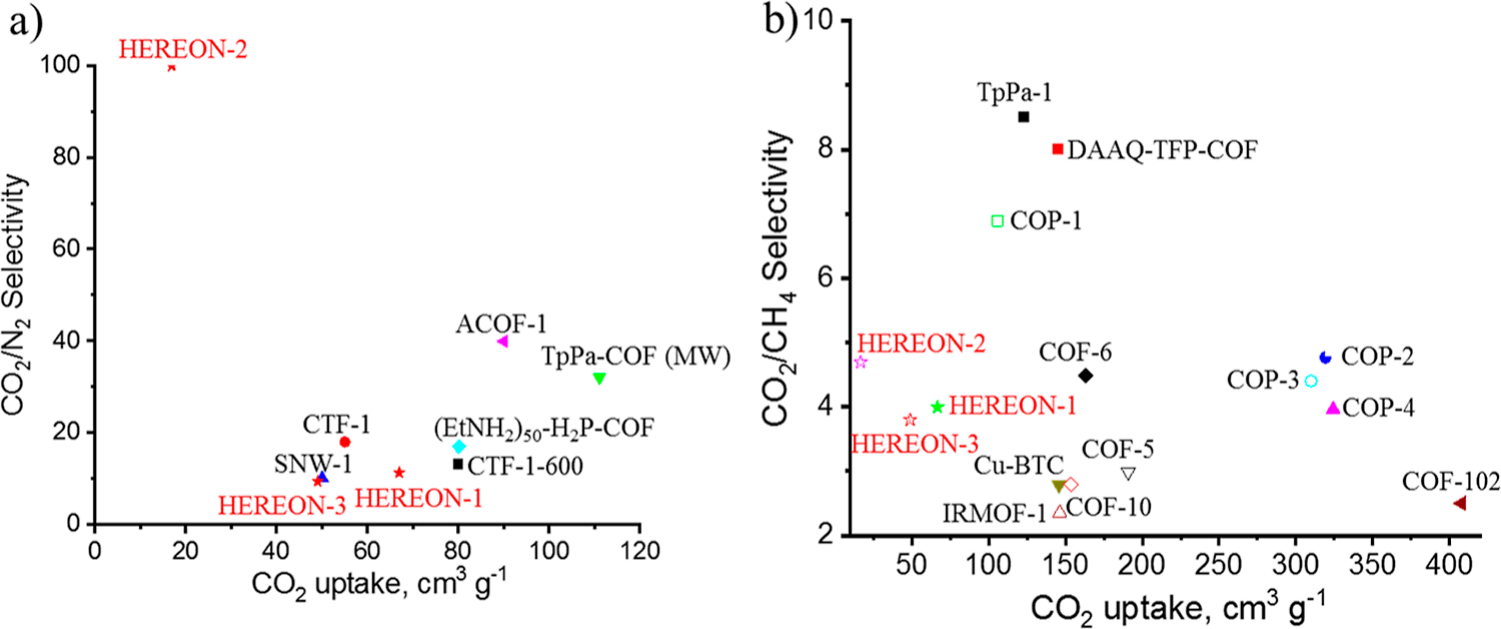
(a) CO_2_ uptake *versus* CO_2_/N_2_ selectivity along with data from,^24^ and (b) CO_2_ uptake *versus* CO_2_/CH_4_ selectivity along with data from refs (74–77).

Considering the results from Figure 8, we claim that the synthesized novel CPNs, especially HEREON2, show much better selectivity compared with the values reported for other COFs having high pore volumes and BET surface areas. The high CO_2_/N_2_ selectivity by HEREON2 is explained by the effect of the pore size and the amount of nitrogen in the structure, in addition to the polarizability and quadrupole momentum. Thus, the triazoles of the ligand contain more nitrogen than other ligands that adsorb more CO_2_ holding them on the adsorbate surfaces and pores. The relationships between the CO_2_/N_2_ and CO_2_/CH_4_ selectivities and the CO_2_ uptake are described in Figure 9.

As seen in Figure 9a, the synthesized CPNs are almost in good agreement with other COFs. Although the CO_2_ uptake is lower in the case of HEREON2, it features high selectivity due to the nature of the synthesized compounds. Furukawa and Yaghi showed that with the increase of pore volume, the amount of guest molecule storage increases as well.^55^ However, the experiments showed that the adsorption (solubility) selectivity of the gas mixtures could be decreased in this case.

The CO_2_/CH_4_ selectivity recorded for the synthesized CPNs is almost the same as the results of other COFs (Figure 9b). Although the CO_2_ uptake is low in the samples, the selectivity could be applicable for the effective separation of CO_2_ from CH_4_.

Based on our results, we will further conduct experiments with the synthesized CPNs, especially synthesizing new materials without side functional groups. Another potential exploration will be the crystallization of such materials in order to fabricate the COFs. Methods for membrane casting from the HEREON2 CPN are under development.

## Conclusions

In this study, three novel CPNs were synthesized, and their structures were characterized by FTIR and SSNMR spectroscopies. XRD and TEM experiments showed imperfections in the crystallinity of the synthesized materials, while TGA results suggest that the synthesized materials are thermally stable. The N_2_ adsorption–desorption experiments showed low BET surface areas and pore volumes, which is related to the nonpolarizability of nitrogen and interparticle aggregation. We suppose that during experiments nitrogen molecules were not polarized by the CPNs, and, for this reason, these compounds demonstrated low surface areas and pore volumes. High-pressure adsorption experiments revealed that our novel CPNs show comparable gas uptakes and CO_2_/N_2_ selectivities compared to the ones in the literature. HEREON2 showed exceptionally high CO_2_/N_2_, and CH_4_/N_2_ selectivities with a solubility separation factor of ∼25,000 and ∼5820. This is directly related to the nonpolarization nature of nitrogen under high pressure in our CPNs.

## Supporting Information Available

Detailed information about experimental procedures for the synthesis of CPNs and their characterization techniques (PDF)
