# Supplementary material for: Two-Dimensional Nanoporous Cross-linked Polymer Networks as Emerging Candidates for Gas Adsorption
Source: ACS Omega. 2024 Mar 22;9(13):15282–93. doi: 10.1021/acsomega.3c09042 (PMC10993420; doi:10.1021/acsomega.3c09042)
Supplement: Supplementary file 1 — ao3c09042_si_001.pdf [file ao3c09042_si_001.pdf]

Supporting Information

TWO-DIMENSIONAL NANOPOROUS CROSSLINKED POLYMER NETWORKS AS EMERGING CANDIDATES FOR GAS ADSORPTION

*Elvin Aliyev[1] †, Thomas Emmler[1], Jelena Lillepaerg[1], Sergey Shishatskiy[1], Nadir Dizge[2], Volkan Filiz[1] **

[1]Institute of Membrane Research, Helmholtz-Zentrum Hereon, Max-Planck Str. 1, 21502 Geesthacht, Germany

[2]Mersin University, Department of Environmental Engineering, 33343 Mersin, Turkey

E-mail: volkan.filiz@hereon.de

Table of Contents

Experimental section…….S2

Results and Discussion………..S3

Experimental section

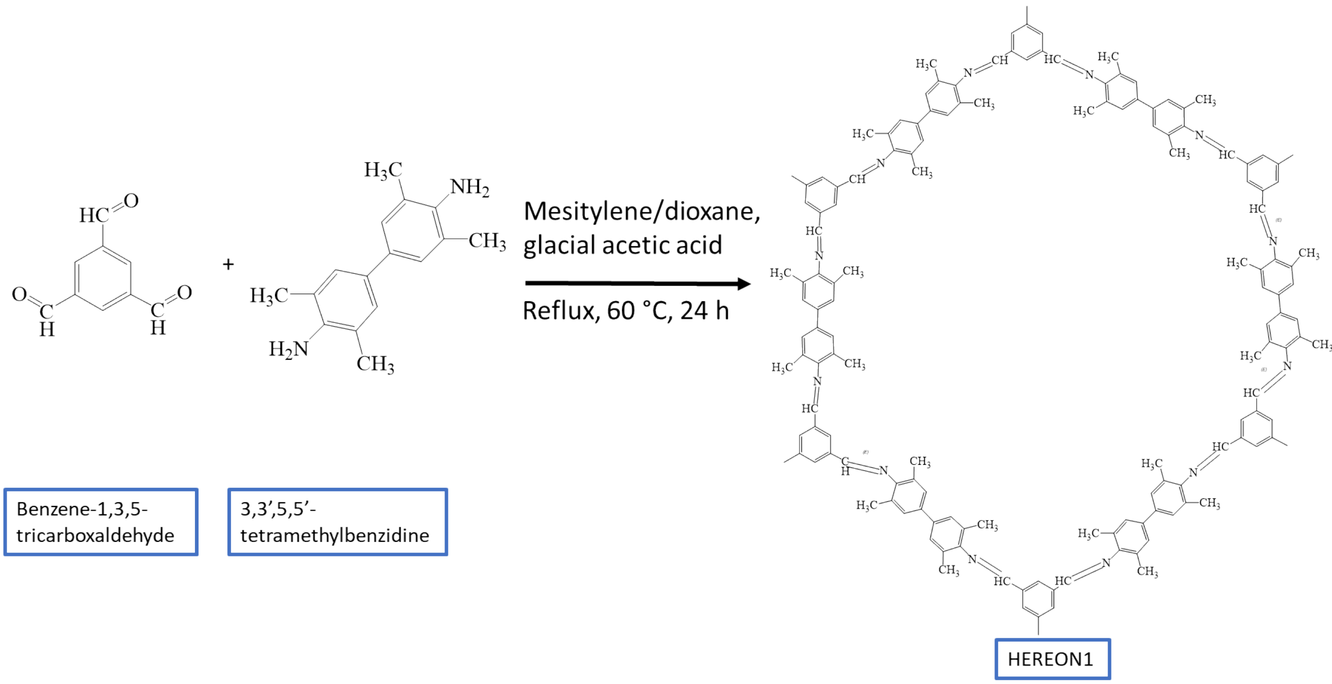

Scheme S1. Synthesis route of HEREON1 comprising benzene-1,3,5-tricarboxaldehyde and 3,3',5,5'-tetramethylbenzidine.

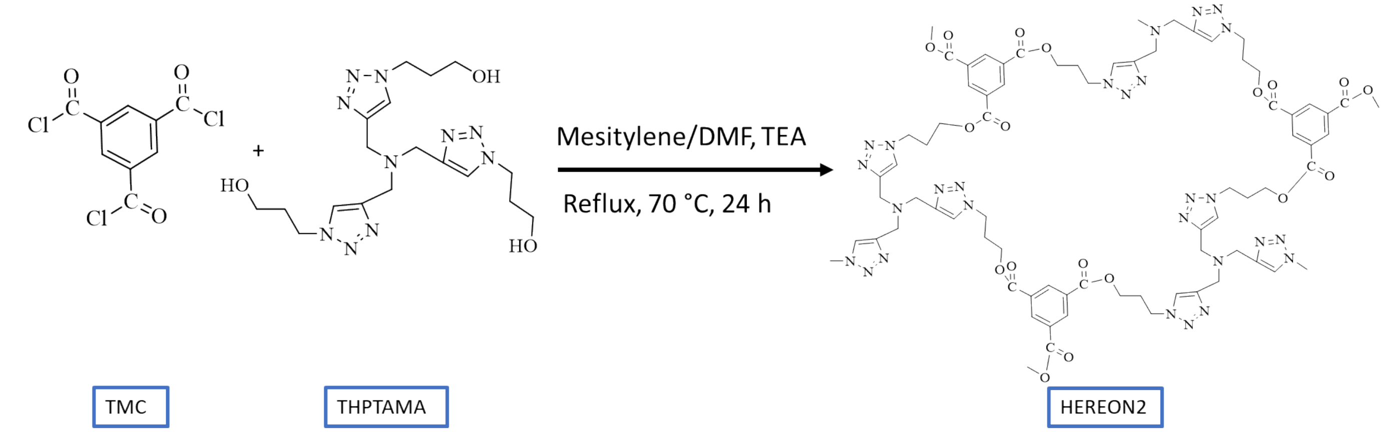

Scheme S2. Synthesis route of HEREON2 comprising 1,3,5-benzenetricarbonyl trichloride (TMC) and tris(3-hydropropyltriazolylmethyl)amine (THPTAMA).

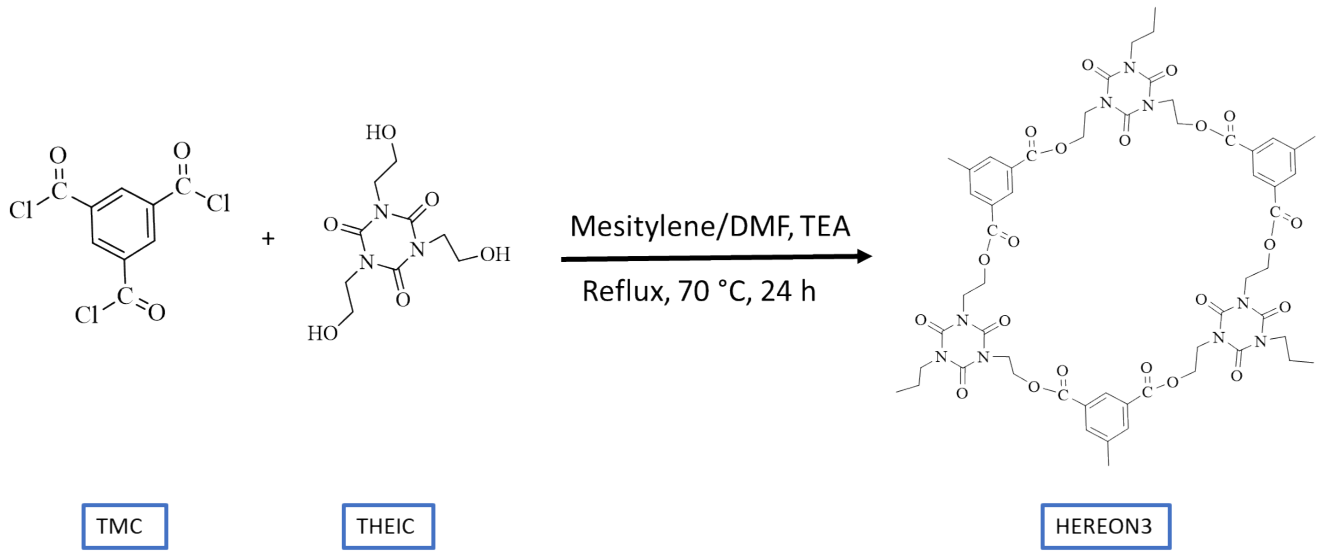

Scheme S3. Synthesis route of HEREON3 comprising 1,3,5-benzenetricarbonyl tricholoride (TMC) and 1,3,5-tris(2-hyroxyethyl)isocyanurate (THEIC).

Results and Discussion

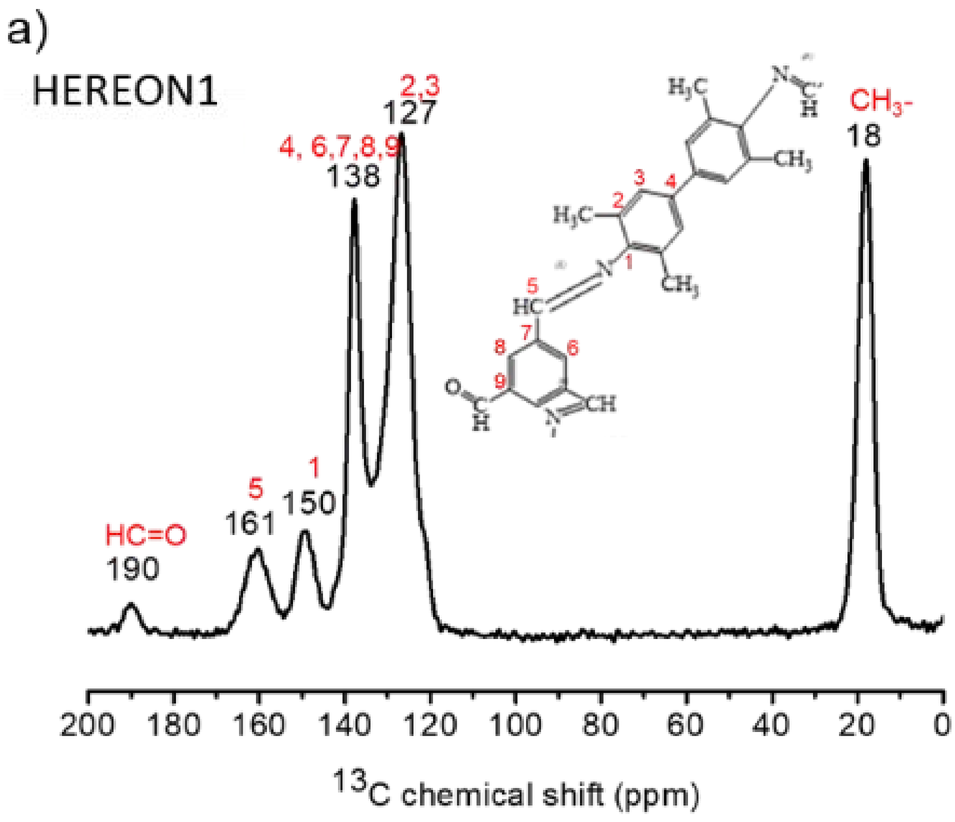

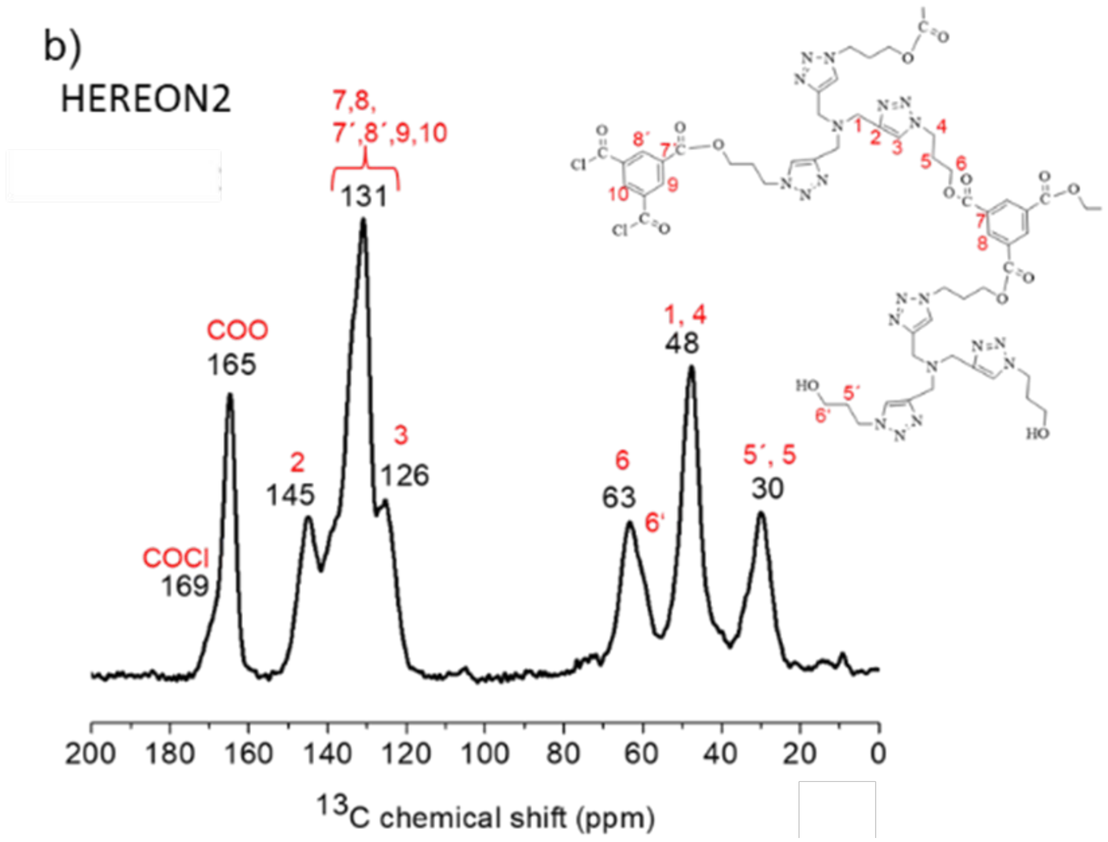
b)
HEREON2
7,8,
7´,8´,9,10
131
COO
165
COCl
169
2
145
3
126
6
63
6'
1, 4
48
5´, 5
30
13C chemical shift (ppm)

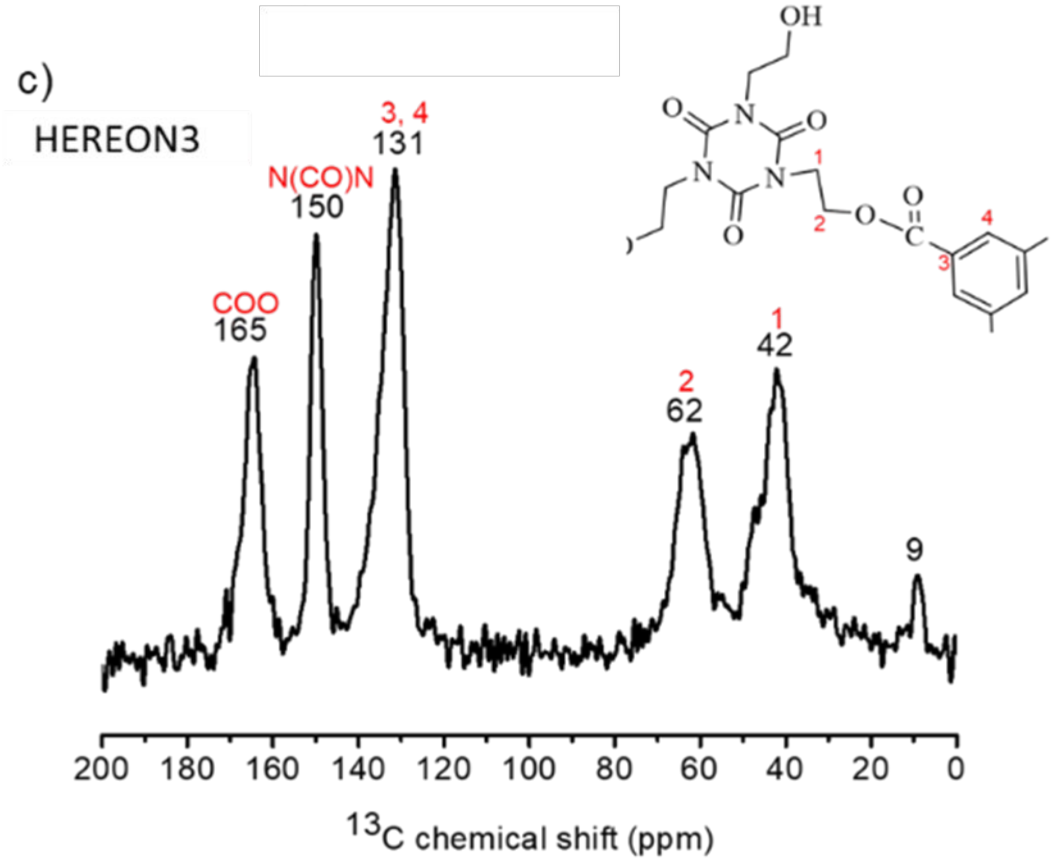
c)
HEREON3
3, 4
131
N(CO)N
150
COO
165
2
62
1
42
9
OH
13C chemical shift (ppm)

Figure 1S. ^{13}C CP MAS NMR analysis of the CPN samples.

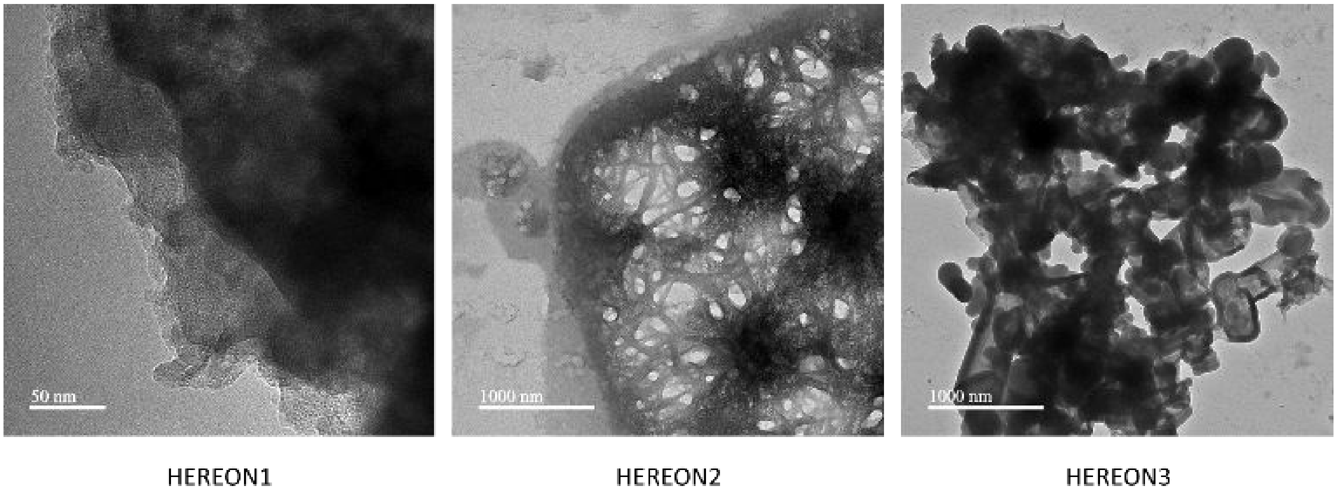

Figure 2S. TEM images of crosslinked polymer networks.

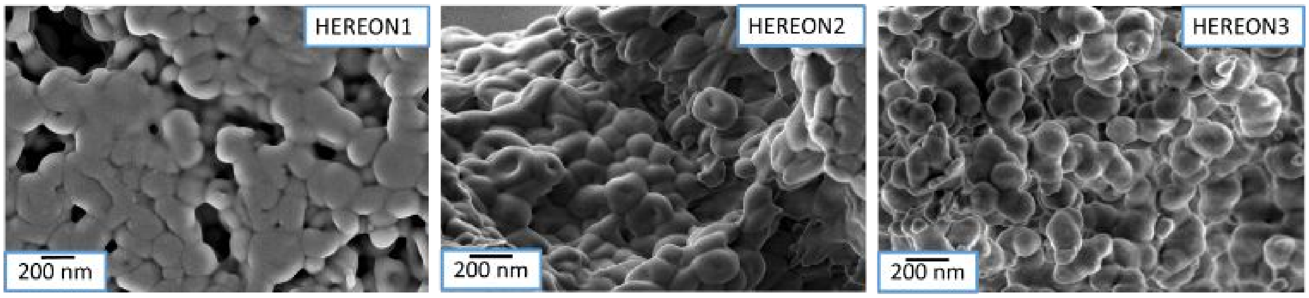

Figure 3S. SEM images of the novel crosslinked polymer networks.
